# The evolutionary history of “suboptimal” migration routes

**DOI:** 10.1016/j.isci.2023.108266

**Published:** 2023-10-20

**Authors:** Staffan Bensch, Violeta Caballero-López, Charlie K. Cornwallis, Kristaps Sokolovskis

**Affiliations:** 1 Molecular Ecology and Evolution Lab, Department of Biology, Lund University, 22362 Lund, Sweden; 2 Department of Biology, University of Turku, Vesilinnantie 5, 20500 Turku, Finland

**Keywords:** Ecology, Ornithology, Evolutionary biology

## Abstract

Migratoriness in birds is evolutionary labile, with many examples of increasing or decreasing migration distances on the timescale of modern ornithology. In contrast, shifts of migration to more nearby wintering grounds seem to be a slow process. We examine the history of how Palearctic migratory landbirds have expanded their wintering ranges to include both tropical Africa and Asia, a process that has involved major shifts in migratory routes. We found that species with shorter migration distances and with resident populations in the Palearctic more often winter in both Africa and Asia. Our results suggest that changes in wintering grounds are not by long-distance migrant populations *per se*, but through historic intermediate populations that were less migratory from which long-distance migration evolved secondarily. The failure of long-distance migrants to shift migration direction to more nearby winter quarters indicates that major modifications to the migratory program may be difficult to evolve.

## Highlights

• Many long distance migrants winter in areas much further away than seems necessary
• We investigate why they have not switched to more nearby wintering sites
• The most extreme migrants appear least likely to switch to new wintering areas
• Historically, new wintering areas were colonized by less migratory populations

## Graphical abstract

**Figure:**
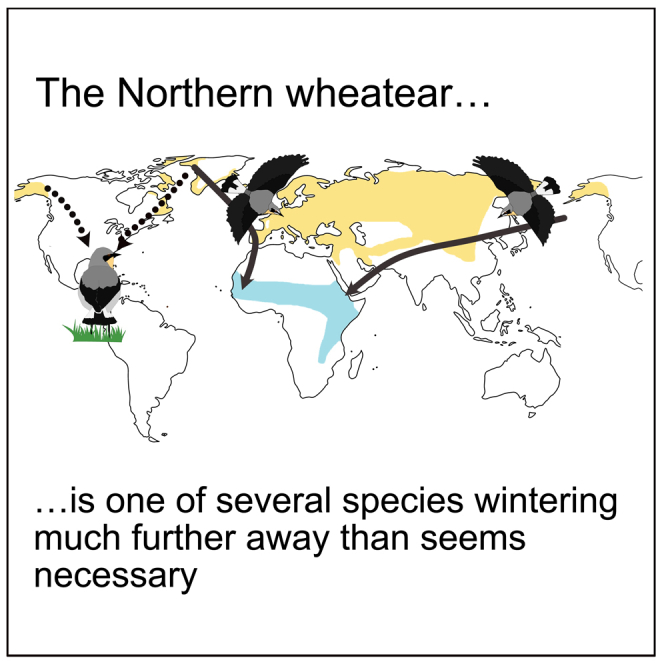

## Introduction

About one-fifth of the world’s bird species are classified as migrants, a trait that has evolved independently multiple times from its first appearance about 80 Mya.^1^ In some lineages, the rate of switching between migration and residency has been so high that it is challenging to reconstruct its evolutionary history.^2^^,^^3^ The plasticity of migration is emphasized by its frequent intra-specific variation; populations of many species are highly divergent in migratory propensity, from fully sedentary to those that migrate between continents. Within migratory species, migration distance typically varies between populations with the northernmost breeding populations often wintering farthest to the south (so called “”leap-frog” migration).^4^^,^^5^ Because the extant breeding ranges of migrants living in the temperate and arctic regions have been colonized since the last glaciation, such population-specific migration distances must have evolved during the Holocene. In contrast to migratory propensity and migration distance that are evolutionary plastic,^6^ there seem to be substantial evolutionary constraints that prevent major switches of migratory direction to novel wintering quarters.^7^ Whether the presumed inability to switch to new wintering grounds is constrained by the genetic migration program *per se*, or by conditions that prevent establishment in potential novel winter quarters (e.g., by novel predators, competitors or pathogens), remains unknown.^8^^,^^9^^,^^10^

In the Palearctic, many strict long-distance migrants with continuous breeding ranges spanning most of the Eurasian landmass have their western populations wintering in Sub-Saharan Africa and their eastern populations wintering in southern Asia (e.g., chiffchaff *Phylloscopus collybita*). Many other species that are equally widely distributed, exclusively winter in either Africa (e.g., willow warbler *Phylloscopus trochilus*) or Asia (e.g., Arctic warbler *Phylloscopus borealis*). It is thought that species that winter exclusively in Africa or Asia have maintained their migratory paths to ancestral wintering grounds during the post-glacial colonization of the present breeding range, but failed to switch to more nearby wintering grounds for some reason. This pattern has been called “suboptimal migration routes” in the literature,^7^^,^^10^^,^^11^^,^^12^^,^^13^ although modeling data suggests that longer migration distances can be energetically optimal when factoring in resource competition, availability of stopover sites and wind assistance.^14^ For the most extreme routes, it is nevertheless hard to envision that these are the most optimal from the perspective of energy efficiency. Striking examples are the northern wheatears *Oenanthe oenanthe* breeding in the Americas and winter in Africa,^13^ and swifts *Apus apus* breeding at the Summer Palace in Beijing and migrating >10,000 km to Africa^15^ even if seemingly similar wintering habitats can be found in southern Asia only ∼3,000 km away.

Here, we examine why some species with wide longitudinal breeding ranges have managed to establish closer wintering ranges, whereas more distant ancestral wintering ranges have been maintained in other species.^7^^,^^11^^,^^16^^,^^17^ Long-distance migrants breeding in the Palearctic are ideal for exploring this question because their tropical winter quarters are in two separate, clearly defined regions, Sub-Saharan Africa and southern Asia. This facilitates the scoring of species that “use” and do “not-use” potential wintering quarters. We assume that species that winter in both Africa and Asia have expanded their tropical wintering range from Africa to Asia or Asia to Africa at some point in their history. In support of this assumption, none of the Palearctic long-distance migrants that have restricted breeding ranges (either in Western Palearctic or Eastern Palearctic) winter in both Africa and Asia. Furthermore, for some species, there is phylogenetic support that they have colonized new winter ranges in Africa or Asia. For example, *Sylvia* warblers originated in the southern part of the Western Palearctic and have ancestral wintering areas in Africa.^18^ It is therefore likely that in the two species (*Sylvia* *curruca* and *S.* *hortensis/crassirostris*) that winter in both Africa and Asia, the Asian wintering ranges evolved secondarily. Similarly, *Locustella naevia* belongs to a clade with Asian long-distance migrants and residents^19^ which makes it likely, from a phylogenetic perspective, that African wintering ranges evolved after those in Asia.

**Figure 1:**
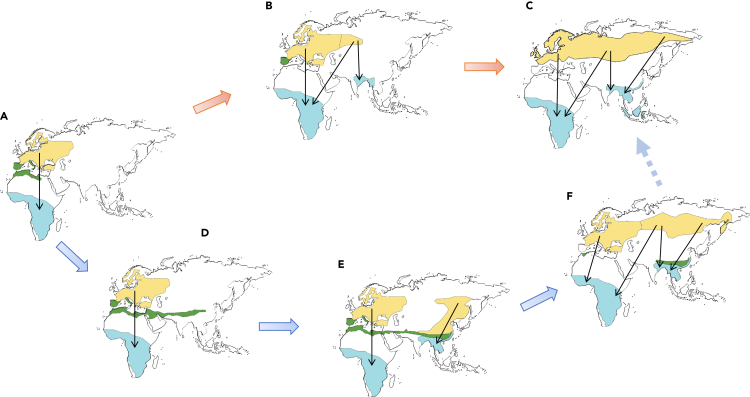
Two graphical models of how long-distance migrants may change wintering continent (A–C) Following a gradually expanding breeding range there is a switch in migratory direction (Model 1). (A, D–F) There is an intermediate phase of sedentary (or short-distance migrants) populations from which long-distance migration evolve secondarily (Model 2). (A) The example shows a species that at some point in history was restricted to the western Palearctic and had both resident (green) and long-distance migratory populations (breeding and winter range in orange and blue). (B) Following expansion of the breeding range, the easternmost population switches migration direction to more nearby winter quarters in southern Asia. (C) The use of new winter grounds facilitates further range expansions eastwards. In this example, the resident populations became extinct. (D) The resident population undergoes a longitudinal range expansion eastward. (E) Migration evolves from the expanded resident population. (F) Further range changes lead to the present distribution of the species.

We propose two different models to explain the process of how long-distance migrants could have evolved novel winter quarters (Figure 1). We illustrate the principles of the models with a species that was initially restricted to the western Palearctic (Figure 1A). Model 1 assumes that the breeding range expanded eastwards and that at some point individuals at the front of the expansion shifted migration toward southern Asia which enabled further expansion of the breeding range (Figures 1A–1C). Model 2 is based on three well-supported observations: (1) Migratory propensity (being a migrant versus a resident) is an evolutionary very labile character state,^2^^,^^6^^,^^20^ (2) migration distance seems to evolve quickly^21^^,^^22^ from partial migrants via short and medium migration distances to long-distance migrants,^23^ and (3) resident and partially migratory species appear to have more rapidly expanded their west-east ranges than long-distance migrants.^24^^,^^25^ In Model 2, sedentary populations (or short-distance migrants) expand their breeding ranges eastwards from which long-distance migrants re-evolve (Figures 1D–1F). We also assume that many of these resident populations that historically have had wider longitudinal distributions that served as dispersal bridges between the Western and Eastern Palearctic might now be extinct.

We examined evidence for models 1 and 2 using 105 species of landbirds breeding in the Palearctic that have some populations wintering in Sub-Saharan Africa. Model 1 predicts that species with more eastern range limits should be more likely to winter in both Africa and Asia. Model 2 makes the additional prediction that long-distance migrants that have resident or wintering populations in the Palearctic are more likely to have established wintering grounds in both Africa and Asia than species that are strictly long-distance migrants.

## Results and discussion

**Figure 2:**
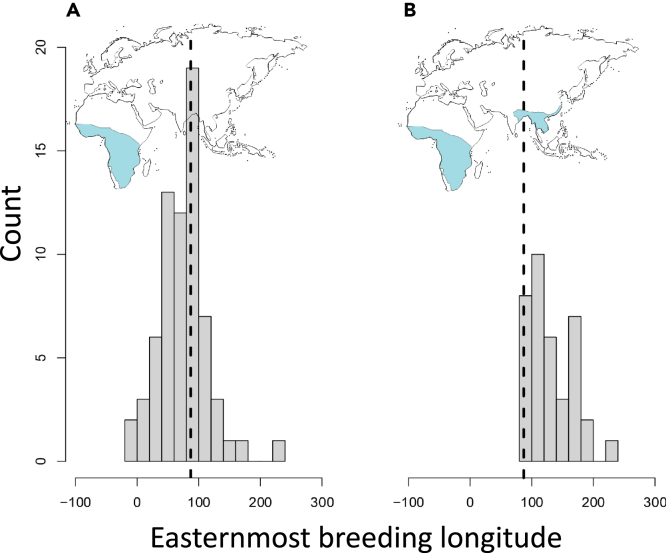
Eastern breeding range limits (longitudes) for long-distance migratory species breeding in the Palearctic that have at least some of their populations wintering in tropical Africa (A) Species with tropical wintering ranges restricted to Africa (N = 66). (B) Species with tropical wintering ranges including both Africa and Asia (N = 39). The stippled vertical line in (A) indicates the longitude of the Yenisey River, from where the distance to the nearest winter grounds in tropical Africa is ∼50% longer than to the nearest winter ground in tropical Asia.

As predicted by both models, species wintering in Africa and Asia have more eastern breeding range limits in the Palearctic than species wintering only in Africa (Bayesian Phylogenetic Mixed Model (BPMM): eastern limit posterior mode (PM) = 2.78, 95% credible interval (CI) = 0.95 to 265.11, pMCMC <0.001. Figure 2, Table S2). Note that several species that migrate only to Africa also breed in the central and eastern Palearctic. From a distance point of view, these species have previously been interpreted as following “sub-optimal” migration routes. For example, 23 strict African migrants (35%) have their breeding ranges extending east of the Yenisey River in Central Asia (Figure 2), and thus migrate a distance ∼50% longer than if they had flown to a wintering area in southern Asia.

**Figure 3:**
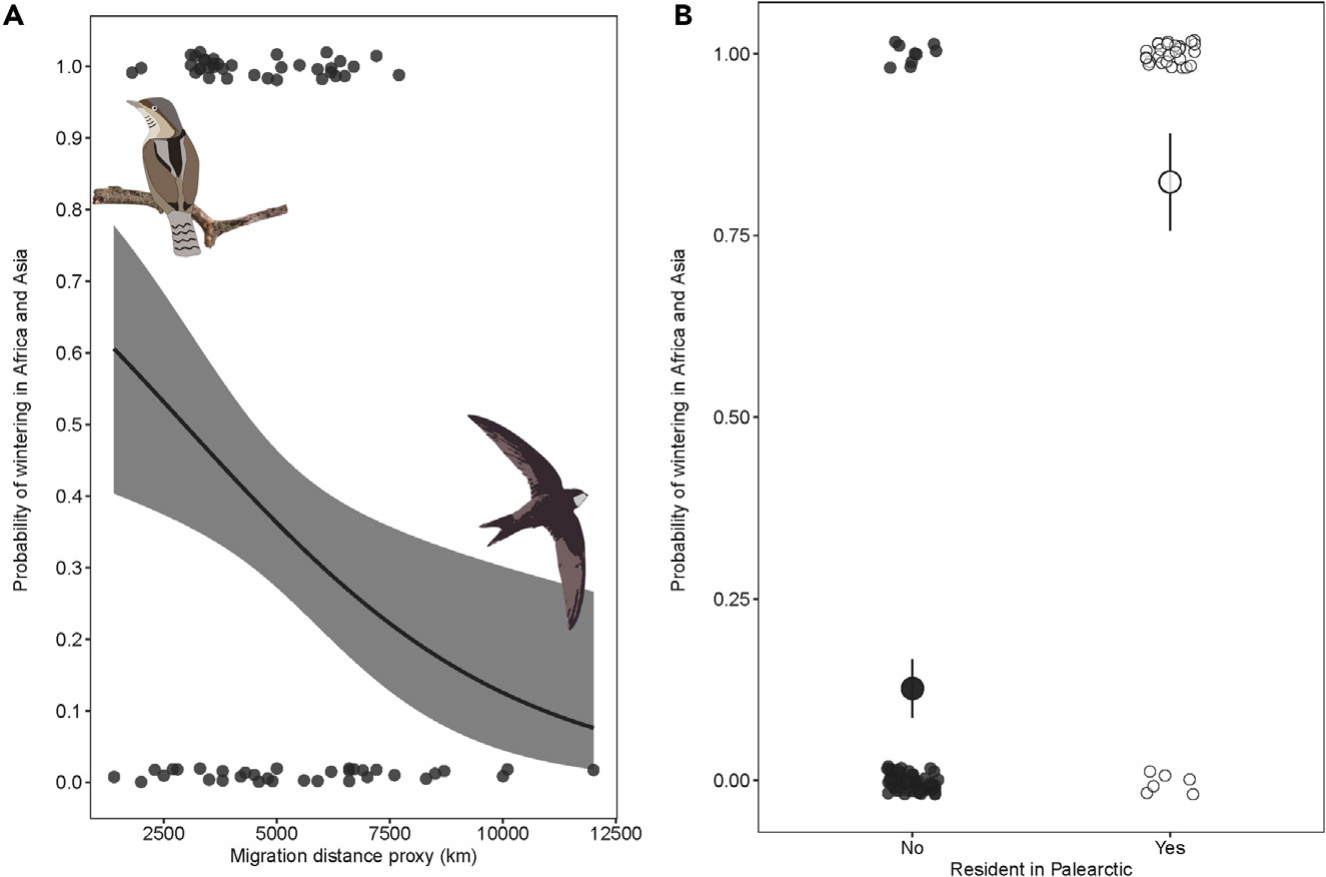
The probability of a species wintering in both Africa and Asia in relation to our proxy of migration distance (MDP) and residency in the Palearctic (ResWin) analyzed using Bayesian Phylogenetic Mixed Models (BPMM) (A) Species with longer migratory distances are less likely (p = 0.001) to evolve wintering ranges in both Africa and Asia. (B) Species that have wintering or resident populations in the Palearctic (open circles) are significantly more likely (p = 0.034) to evolve migratory routes to both Africa and Asia compared to species that exclusively winter in sub-Saharan Africa (black circles). Error bars correspond to SEM.

Next, we investigated whether two measures of the species’ migratoriness (Figure S1) were related to wintering only in Africa versus wintering in Africa and Asia. The first variable, ResWin (Res-ident or Win-tering in the Palearctic), was assigned 0 for strict long-distance migrants exclusively wintering in the tropics and 1 for species having resident or wintering populations in the Palearctic. The second variable, MDP (Migration Distance Proxy), is an indicator of the species’ long-distance migratory capacity, measured as the distance that must be traveled by the individuals that occupy the breeding areas furthest from wintering area (Figures S1 and S2). In support of Model 2, we find that species that winter in both Africa and Asia more often have populations that are less migratory (BPMM of ResWin: PM = 3.38, CI = −5.51 to 218.00, pMCMC = 0.038) and migrate shorter distances (BPMM of MDP: PM = −187.43, CI = −271.09 to −2.93, pMCMC <0.001) than species wintering only in Africa (Figure 3; Table S3). A higher proportion of non-passerines (52%) winter in both Africa and Asia than passerines (28%) (Table 1), but in both groups the traits are broadly distributed across the phylogeny (Figure S3). Likewise, the proxies for migratoriness (ResWin and MPD) are also scattered across the phylogeny (Figure S3), with estimates of the proportion of variation in the probability of migrating to Africa versus to Africa and Asia explained by phylogenetic history being highly variable (PM = 37.48, CI = 0 to 99. Table S3).

**Table 1:** Number of long-distance migratory bird species wintering in tropical Africa versus also in southern Asia, in relation to whether they have some populations that are resident or wintering in the Palearctic

	Tropical Winter Range	Resident/wintering Palearctic		Total
		No	Yes	
Non-Passerines:	Africa	17	3	20
	Africa & Asia	5	13	18
Passerines:	Africa	46	2	48
	Africa & Asia	5	14	19
Total		73	32	105

Intuitively, one may expect that species with the longest migration distance would be most capable of adding a new continent to their wintering range because they already travel the world. In contrast, we find that our measure of migration distance proxy (MDP) is negatively correlated to the probability of the species wintering in both Africa and Asia. Evolutionary constraints on establishing novel winter quarters may therefore explain why long-distance migrants have more restricted breeding ranges longitudinally than sympatrically breeding resident bird species.^24^^,^^25^ Resident species can expand their ranges west-east without novel genetic adaptations, as long as the climate and habitat are suitable. Conversely, for long-distance migrants to reach their wintering grounds, modifications to genetic mechanisms controlling migration may be required, which may slow down the west-east range expansion.^24^^,^^26^^,^^27^ In line with this idea, species distribution models suggest that wintering ranges of West Palearctic long-distance migrants were relatively similar at the last glacial maximum compared to present, whereas breeding areas were substantially contracted with northern and eastern range limits displaced southward and westwards, respectively.^28^

It may seem counterintuitive that the most extreme migrants are those that are most unlikely to establish new winter continents, but suggests that the genetic migration program may be evolutionary difficult to modify.^29^ Still, winter continent changes do take place, since about one-third of the long-distance migrants winter in both Africa and Asia. The relationship can be resolved by assuming that these changes in wintering grounds were not by the long-distance migrant populations *per se* (Model 1), but through a series of historically intermediate populations that were less migratory (Model 2). If long-distance migratory species presently wintering both in Africa and Asia were historically connected with resident or short-distance migrant populations that expanded longitudinally, long-distance migration may have evolved secondarily (Figure 1).

Presently, only a few of the species wintering in both tropical Africa and Asia (e.g., peregrine falcon *Falco peregrinus*) have resident or short-distance migrants continuously distributed throughout Eurasia. Most have one, or a few, geographically restricted resident or short-distance migratory populations, typically located in the Saharo-Arabian and the Sino-Japanese zoogeographic regions,^30^ such as the wryneck *Jynx torquilla* (Figure S1B). We suggest that these are remnants of formerly longitudinal widespread and less migratory populations that thrived during the Pleistocene, when the Saharo-Arabian and the Sino-Japanese regions were wetter and less dominated by deserts. Presumably, such conditions could have supported resident and wintering populations of long-distance migrants. Such a scenario of historically widespread populations has been suggested to explain the disjunct breeding range (SW Europe and E Asia) of the sedentary azure-winged magpie *Cyanopica cyanus*.^31^^,^^32^ We therefore propose that major wintering ground expansions of long-distance migratory species are typically not from their long-distance migration populations, but evolved secondarily from historically widespread less migratory populations, some of which might now be extinct.

Our proposed Model 2 can be tested by using phylogenetic analyses of populations within species that migrate to tropical Africa and Asia that have resident or short-distance populations present in Eurasia. The prediction is that the long-distance migratory populations should not be their closest relatives; one should be more closely related to a less migratory population (Figure S4). Sequence-based phylogenies covering both long-distance and resident/short-distance populations remain to be obtained for the species in the present study, with the exception of the common chiffchaff *P. collybita*.^33^ In support of the Model 2, the long-distance migratory Siberian *P.* *tristis* (wintering in southern Asia) and the European *P. collybita* (West Africa) and *P*. *abietinus* (East Africa) are not their closest relatives; they are nested in between the resident or short-distance migrants *P*. *brevirostris* and *P*. *caucasicus*.

The present study focuses on the migratory flyways of the Old World, but the principles we are investigating are general and should apply also to the migration system in America. The wintering grounds in the New World are much more compressed longitudinally than in the Old World with less clear distinctions between “used” and “not-used” wintering grounds, which will be a challenge for the analyses. However, a prediction from Model 2 is supported by phylogeographic analyses of two North American migrants. In common yellowthroats *Geothlypis trichas*, eastern and western long-distance migratory populations are more closely related to southern residents than to each other.^34^ Similarly, in the yellow-rumped warbler species complex (*Setophaga* spp.), the migratory *Setophaga auduboni* that winters in the west is more closely related to the resident *Setophaga audoboni nigrifons* than to the migratory *Setophaga coronata* that winters in the east.^35^^,^^36^

The results we present predict that shifting between major wintering quarters is a slow process since it requires an intermediate step of historical populations that were sedentary or short-distance migrants. This suggests that long-distance migrants wintering in Africa and Asia probably diverged during, or before, the last glaciation (20 to several 100 kya), with few if any shifts during the Holocene (<12 kya). Ten of the species with populations wintering in both Africa and Asia have clearly separated subspecies suggesting that they diverged a long time ago, but direct estimates exist only for the chiffchaff: *collybita* and *abietinus* wintering in Africa and *tristis* in Asia that diverged ∼0.25 Mya.^33^ However, the majority are either monotypic (eight species) or have the same subspecies recorded as wintering in both Africa and Asia (19 species) (Table S1). Investigating these species by time-calibrated phylogenetic analyses will reveal whether winter continent expansion is primarily a slow process that requires intermediate steps of less migratory populations, as suggested by Model 2, or if some shifts also have taken place during Holocene (Model 1).

Four of the long-distance migrants wintering in both Africa and Asia also have long-distance migratory populations breeding in North America and wintering in Central or South America (peregrine falcon, osprey *Pandion haliaetus*, barn swallow *Hirundo rustica* and sand martin *Riparia riparia*). As predicted by Model 2, all of these species have sedentary populations somewhere within their ranges. Estimates of divergence times between the Eurasian and American long-distance migrants is available for two of these species; ospreys in the Old and New World diverged 1.1 Mya^37^ and barn swallows 50–100 kya.^38^^,^^39^ These divergence times are in line with our prediction that major changes in wintering areas were not made by long-distance migratory populations during Holocene.

Our analyses imply that major changes to wintering areas occur over long time scales, with the migration routes of many species traceable back at least to the last ice age. But how do we reconcile this apparent inertia with the recent changes in migration direction reported for several birds? Two Siberian species that winter in southern Asia seem to have an increasing fraction of their populations migrating to winter quarters in western Europe.^40^^,^^41^ It remains, however, to be demonstrated whether this is a new phenomenon, as very low numbers of Siberian birds migrating to Europe might have passed undetected until the present era of modern field ornithology. A well-established textbook example of a recent change in migration direction is the central European blackcaps, *Sylvia atricapilla,* a short-distance migrant that has started to winter on the British Isles rather than in the Mediterranean area, a shift of autumn migration direction from SW to NW.^42^ Light-level geolocator tracks of blackcaps^43^ have shown that the “new” NW migratory phenotype is not localized to a narrow region in central Europe as previously thought,^44^^,^^45^ but occurs widely and at low frequencies within its breeding range from Spain to Poland. Hence, the mechanism underlying the wide geographic distribution of this phenotype is of a much older date than the <100 years previously suggested.

Shifts of migration routes to a novel wintering continent probably require substantial changes in the migratory program, whereas in blackcaps, it may only involve a change in direction. This is because shifts to novel and distant winter quarters also require adjustments of stopover sites relative to barrier crossings and sometimes detours to avoid, or take advantage of, prevailing wind directions.^46^ The fact that long-distance migrants seem stuck with their ancestral migration routes and wintering quarters, whereas short-distance migrants are more flexible, such as the blackcap that have changed migration direction quite recently, may not be as paradoxical as it first seems.

Many species of long-distance migrants are rapidly declining, most likely as a consequence of ongoing global change.^47^ From an evolutionary perspective, an interesting implication of Model 2 is that resident populations, in otherwise migratory species, may have an overlooked conservation value, since these seem to have the capacity to re-evolve the migratory behavior rather easily if the former become extinct.

### Limitations of the study

Our analyses suggest that the migratory program of long-distance migrants is difficult to change. However, this conclusion is based on indirect evidence that will require further testing. We propose that future research should make use of the steadily increasing tracking data to investigate whether short-distance migrants have a more flexible migratory program than long-distance migrants.

## STAR★Methods

### Key resources table

**Table:** 

REAGENT or RESOURCE	SOURCE	IDENTIFIER
**Software and algorithms**		

R package MCMCglmm	Hadfield (2010)^48^	https://doi.org/10.18637/jss.v033.i02
R package ‘coda’	Plummer et al. (2006)^49^	https://journal.r-project.org/articles/RN-2006-002/RN-2006-002.pdf

**Other**		

Birdtree phylogeny	Jetz et al. (2012)^50^	https://birdtree.org/
Birds of the World	del Hoyo (2020)^51^	https://birdsoftheworld.org/bow/home

### Resource availability

#### Lead contact

Further information and requests should be directed to and will be fulfilled by the lead contact, Staffan Bensch (staffan.bensch@biol.lu.se)

#### Materials availability

This study did not generate new materials.

### Experimental model and study participant details

We selected all species of landbirds that have populations that are long-distance migrants and use clearly defined tropical wintering areas on one (Africa) or two separate (Africa and Asia) continents. The species we included had to be breeding in the Palearctic and therefore have some populations crossing major ecological barriers (vast deserts and water bodies) for wintering in sub-Saharan Africa. We used this approach to identify long-distance migrants as it is a less arbitrary way than by a chosen distance (e.g., 2,000 km). To control for shared evolutionary history in the analyses, we used the phylogeny of^50^ downloaded from www.birdtree.org. A few taxa, presently considered to be different species in the IOC World Bird List (https://www.worldbirdnames.org/new/), were therefore treated as subspecies and included as members of a species present in the birdtree dataset (Table S1). Of the long-distance migratory landbirds breeding in Palaearctic,^51^ 68 were classified as having tropical winter ranges in Sub-Saharan Africa only, whereas 39 were scored to winter also in southern Asia (Table 1). We assume that species that winter in both Africa and Asia, have expanded their tropical wintering range (from Africa to Asia or Asia to Africa) at some point in history. To extract information about migration distance, breeding and wintering ranges and presence of resident populations (Figure S1), we used the distribution maps in del Hoyo (2020)^51^ and corroborated details by consulting additional sources.^52^^,^^53^

### Method details

#### Estimates of migratoriness

Breeding and wintering ranges were quantified by extracting the northernmost, southernmost, westernmost and easternmost latitudes and longitudes, respectively. We created two variables “ResWin” and “MDP”, that can be thought of as proxies for the species migratoriness. The variable ResWin (Res-ident or Win-tering in the Palearctic) is assigned 0 for strict long-distance migrants exclusively wintering in the tropics and 1 for species having resident populations or wintering populations in the Palearctic. The variable MDP (Migration Distance Proxy) is the longest distance from a breeding area to the wintering area. This migration distance variable assumes that the birds are using the closest wintering area which in many cases is not correct. However, these are conservative estimates that can be objectively quantified in all species in the absence of direct measures of migration distances, which are only available for a restricted number of species. In support of this approach, in 36 species for which migration distances have been obtained by satellite tracking or light-level geolocators, MDP is strongly correlated to the tracked distance (Figure S2). All data used in analyses are presented in Table S1.

### Quantification and statistical analysis

Data were analyzed using Bayesian Phylogenetic Mixed Models (BPMM) with Markov chain Monte Carlo (MCMC) estimation in the R package MCMCglmm.^48^ First, we examined if species with more eastern breeding ranges (continuous fixed effect) had a higher probability of wintering in Asia as well as Africa (1,0), modeled as a binary response variable with a logit link function (Table S2). Second, we tested if species with resident (or short-distance migrant) breeding populations (two-level fixed factor) differed in their probability of migrating to Africa and Asia (1,0). The effect of breeding range distribution on probability of migrating to Africa and Asia was controlled for by fitting western and eastern longitude, and southern latitude of breeding ranges as continuous fixed effects (Table S3). Continuous explanatory variables were Z-transformed prior to analyses (mean = 0, standard deviation = 1). Northern latitude was not included in models as it was strongly correlated to eastern longitude (r = 0.7). However, we verified that our results were not dependent upon on excluding northern latitude by re-running models with northern latitude included instead of eastern longitude (Table S4). We recovered quantitatively similar results regardless of which breeding range variables were included.

The non-independence of data resulting from phylogenetic relatedness between species was modeled by fitting a phylogenetic variance-co-variance matrix constructed from the birdtree phylogeny (Figure S3).^50^ To account for phylogenetic uncertainty, we ran models across a sample of 1500 trees. Estimates from the last iteration from tree *i* were used to as starting parameter values for tree *i*+1. Estimates from the last iteration of each tree were saved, with samples from the first 500 trees being discarded as a burn-in. Each tree was sampled for 10000 iterations with only the last iteration being saved resulting in a posterior distribution of 1000 samples for parameter estimation.

Parameter estimates from models are presented as posterior modes (PM) with 95% credible intervals (CIs). p values (pMCMC) were estimated as the number of posterior samples above or below a specified value divided by the total number of posterior samples, corrected for the finite number of MCMC samples.^48^ We estimated the amount of variation in the probability that species migrate to Africa and Asia explained by phylogeny using the intraclass correlation coefficient (ICC) calculated on the latent scale as:

$$V_{i}\;/\;V_{\text{RE}}+V_{e}$$

where V_i_ is the focal random effect, V_RE_ is the sum of all random effects and V_e_ is the residual variance on the latent scale. For binary traits, the residual variance is unidentifiable and was fixed to 1. Ve on the latent scale was therefore calculated as the residual variance on the logit scale (1) plus the variance associated with the link function (logit = $i^{2/3}$ . See^54^; ^55^ for discussion).

#### Prior settings and model convergence

For fixed effects, a prior of mu = 0, V = σ2 units + $\pi^{2/3}$ was specified. This is approximately flat on the probability scale when a logit link function is defined.^48^ For random effects, we used inverse-Wishart priors (V = 1, nu = 0.002) and the residual variance was fixed to 1. We examined the model convergence by repeating each analysis three times and examining the correspondence between chains using the R package ‘coda’^49^ in the following ways: (i) visually inspecting the traces of the MCMC posterior estimates and their overlap; (ii) calculating the autocorrelation and effective sample size of the posterior distribution of each chain; and (iii) using Gelman and Rubin’s convergence diagnostic test that compares within- and between-chain variance using a potential scale reduction factor (PSR). PSR values substantially higher than 1.1 indicate chains with poor convergence properties.^56^ For details of all analyses see Supplementary R code (See R script “Analyses.R”). All analyses were conducted in R 4.1.^57^

## Data Availability

• All data extracted from open databases and publications are available in Table S1 (LINK to be added). • The code used for analysing the data is provided in supplemental information under the heading “Analyses.R” (LINK to be added). • Any additional information required to reanalyze the the data reported in this paper is available from the lead contact upon request.
