# Supplementary material for: The evolutionary history of “suboptimal” migration routes
Source: iScience. 2023 Oct 20;26(11):108266. doi: 10.1016/j.isci.2023.108266 (PMC10663737; doi:10.1016/j.isci.2023.108266)
Supplement: Document S1. Figures S1–S4 and Tables S1–S4 [file mmc1.pdf]

iScience, Volume *26*

Supplemental information

The evolutionary history of “suboptimal” migration routes

Staffan Bensch, Violeta Caballero-López, Charlie K. Cornwallis, and Kristaps Sokolovskis

Supplemental Information

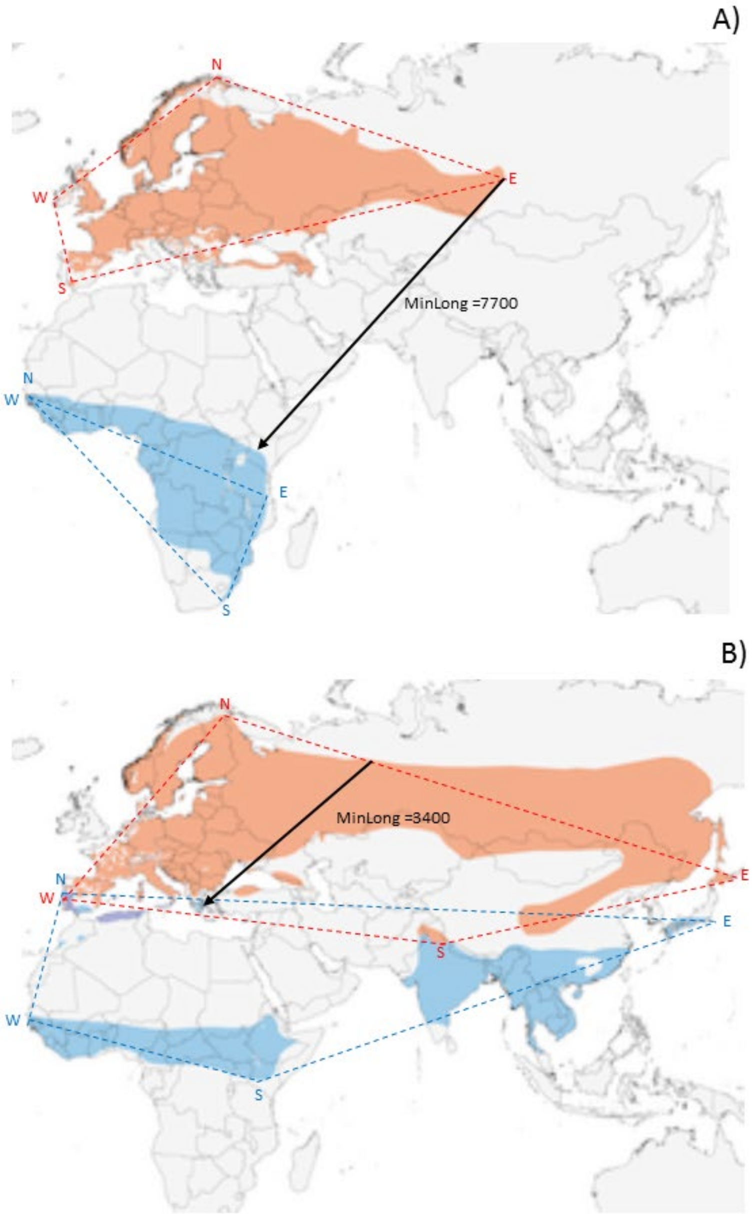

Figure S1. **Two examples of data extracted from distribution maps. Related to STAR Methods.** Distribution maps of all species were scored for endpoints of breeding (red) and winter ranges (blue) and estimates of the species minimum migration distance capacity in km (MDP – Migration Distance Proxy), i.e. the distance furthest from the wintering area. A. Garden warbler *Sylvia borin* and B. wryneck *Jynx torquilla*. The garden warbler is a strict long distance migratory species (ResWin = 0) whereas the wryneck has resident (North Africa) and wintering populations (Japan) within the Palearctic (ResWin = 1).

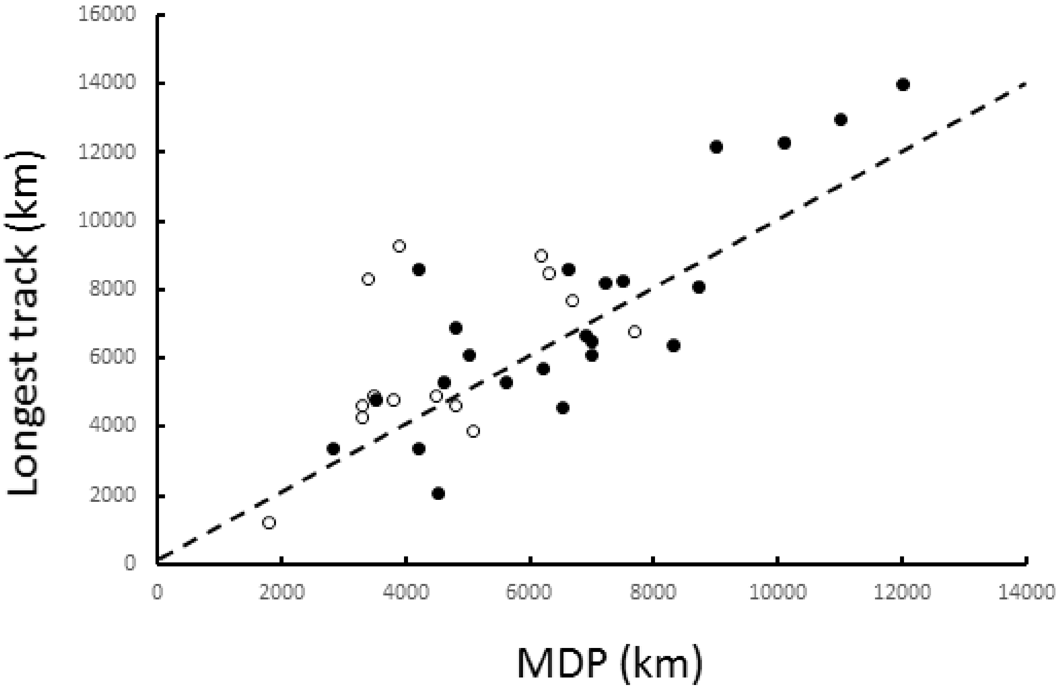

Figure S2. Data supporting the use of a proxy for measuring the species migration distance (MPD). Related to STAR Methods. Correlation between our proxy for migration distance (MDP) and the longest tracked distance (satellite or geolocators) between breeding and winter quarters (37 species). Species only wintering in Africa are represented by closed circles (r = 0.84, P < 0.001, n = 23) and those wintering both Africa and Asia by open circles (r = 0.56, P = 0.035, n = 14). The stippled line shows Y = X.

Species included in the analyses with references to the publications: Buteo buteo, [1] Circaetus gallicus,[2] Circus aeruginosus, [3] Circus pygargus,[4, 5] Aquila pomarine,[6] Milvus migrans,[7, 8] Pernis apivorus,[9] Pandion haliaetus,[10] Apus apus,[11, 12] Apus pallidus,[13] Tachymarptis melba,[14] Apus caffer,[15] Caprimulgus europaeus,[16, 17] Upupa epops,[18] Cuculus canorus,[19, 20] Falco Subbuteo,[21] Falco eleonorae,[22] Falco concolor,[23] Falco peregrinus,[24] Hirundo daurica, [25] Hirundo rustica,[26, 27] Erythropygia galactotes,[15] Luscinia svecica,[15, 28, 29] Ficedula albicollis,[30] Ficedula hypoleuca,[31, 32] Ficedula semitorquata,[33] Luscinia Luscinia,[34] Luscinia megarhynchos,[35] Oenanthe Oenanthe,[36] Sylvia atricapilla,[37, 38] Acrocephalus arundinaceus,[39] Acrocephalus scirpaceus,[40] Acrocephalus paludicola,[41] Phylloscopus sibilatrix,[42] Phylloscopus trochilus,[43, 44] Lanius collurio,[45, 46] Emberiza hortulana,[47].

1. Strandberg, R., Alerstam, T., Hake, M., and Kjellén, N. (2009). Short-distance migration of the Common Buzzard Buteo buteo recorded by satellite tracking. Ibis *151*, 200-206. https://doi.org/10.1111/j.1474-919X.2008.00890.x
2. Mellone, U., Lucia, G., Mallìa, E., Urios, V., and Arroyo, B. (2016). Individual variation in orientation promotes a 3000-km latitudinal change in wintering grounds in a long-distance migratory raptor. Ibis *158*, 887-893. https://doi.org/10.1111/ibi.12401

3. Vansteelant, W.M.G., Klaassen, R., Strandberg, R., Janssens, K., T´Jollyn, F., Bouten, W., Koks, B.J., and Anselin, A. (2020). Western Marsh Harriers Circus aeruginosus from nearby breeding areas migrate along comparable loops, but on contrasting schedules in the West African–Eurasian flyway. J. Ornith. *161*, 953-965. https://doi.org/10.1007/s10336-020-01785-6
4. Limiñana, R., Soutullo, A., Urios, V., and Reig-Ferrer, A. (2011). Migration and wintering areas of adult Montagu's Harriers (Circus pygargus) breeding in Spain. J. Ornith. *153*, 85-93. https://doi.org/10.1007/s10336-011-0698-x
5. Trierweiler, C., Klaassen, R.H., Drent, R.H., Exo, K.M., Komdeur, J., Bairlein, F., and Koks, B.J. (2014). Migratory connectivity and population-specific migration routes in a long-distance migratory bird. Proc. Biol. Sci. *281*, 20132897. https://doi.org/10.1098/rspb.2013.2897
6. Väli, U., Mirski, P., Sellis, U., Dagys, M., and Maciorowski, G. (2018). Genetic determination of migration strategies in large soaring birds: evidence from hybrid eagles. Proc. Biol. Sci. *285*. https://doi.org/10.1098/rspb.2018.0855
7. Sergio, F., Tanferna, A., De Stephanis, R., Jimenez, L.L., Blas, J., Tavecchia, G., Preatoni, D., and Hiraldo, F. (2014). Individual improvements and selective mortality shape lifelong migratory performance. Nature *515*, 410-413. https://doi.org/10.1038/nature13696
8. Literak, I., Skrabal, J., Karyakin, I.V., Andreyenkova, N.G., and Vazhov, S.V. (2022). Black Kites on a flyway between Western Siberia and the Indian Subcontinent. Sci. Rep. *12*, 5581. https://doi.org/10.1038/s41598-022-09246-1
9. Vansteelant, W.M.G., Kekkonen, J., and Byholm, P. (2017). Wind conditions and geography shape the first outbound migration of juvenile honey buzzards and their distribution across sub-Saharan Africa. Proc. Biol. Sci. *284*. https://doi.org/10.1098/rspb.2017.0387
10. Hake, M., Kjellén, N., and Alerstam, T. (2001). Satellite tracking of Swedish Ospreys Pandion haliaetus: autumn migration routes and orientation. J. Avian Biol. *32*, 47-56. https://doi.org/10.1034/j.1600-048X.2001.320107.x
11. Åkesson, S., Atkinson, P.W., Bermejo, A., de la Puente, J., Ferri, M., Hewson, C.M., Holmgren, J., Kaiser, E., Kearsley, L., Klaassen, R.H.G., et al. (2020). Evolution of chain migration in an aerial insectivorous bird, the common swift Apus apus. Evolution *74*, 2377-2391. https://doi.org/10.1111/evo.14093
12. Zhao, Y., Zhao, X., Wu, L., Mu, T., Yu, F., Kearsley, L., Liang, X., Fu, J., Hou, X., Peng, P., et al. (2022). A 30,000-km journey by Apus apus pekinensis tracks arid lands between northern China and south-western Africa. Mov. Ecol. *10*, 29. https://doi.org/10.1186/s40462-022-00329-2
13. Norevik, G., Boano, G., Hedenström, A., Lardelli, R., Liechti, F., and Åkesson, S. (2018). Highly mobile insectivorous swifts perform multiple intra-tropical migrations to exploit an asynchronous African phenology. Oikos *128*, 640-648. https://doi.org/10.1111/oik.05531
14. Meier, C.M., Karaardıç, H., Aymí, R., Peev, S.G., Witvliet, W., and Liechti, F. (2020). Population-specific adjustment of the annual cycle in a super-swift trans-Saharan migrant. J. Avian Biol. *51*. https://doi.org/10.1111/jav.02515
15. Vega, M.L., Willemoes, M., Arizaga, J., Onrubia, A., Cuenca, D., Alonso, D., Torralvo, C., Tøttrup, A.P., and Thorup, K. (2019). Migration strategies of Iberian breeding white-rumped swifts Apus caffer, rufous-tailed scrub-robins Cercotrichas galactotes and bluethroats Cyanecula svecica. Ardeola *66*. https://doi.org/10.13157/arla.66.1.2019.ra4
16. Evens, R., Kowalczyk, C., Norevik, G., Ulenaers, E., Davaasuren, B., Bayargur, S., Artois, T., Åkesson, S., Hedenstrom, A., Liechti, F., et al. (2020). Lunar synchronization of daily activity patterns in a crepuscular avian insectivore. Ecol. Evol. *10*, 7106-7116. https://doi.org/10.1002/ece3.6412
17. Norevik, G., Åkesson, S., Artois, T., Beenaerts, N., Conway, G., Cresswell, B., Evens, R., Henderson, I., Jiguet, F., and Hedenström, A. (2020). Wind-associated detours promote seasonal migratory

connectivity in a flapping flying long-distance avian migrant. J. Anim. Ecol. *89*, 635-646. https://doi.org/10.1111/1365-2656.13112
18. van Wijk, R.E., Schaub, M., Hahn, S., Juarez-Garcia-Pelayo, N., Schafer, B., Viktora, L., Martin-Vivaldi, M., Zischewski, M., and Bauer, S. (2018). Diverse migration strategies in hoopoes (Upupa epops) lead to weak spatial but strong temporal connectivity. Naturwissenschaften *105*, 42. https://doi.org/10.1007/s00114-018-1566-9
19. Willemoes, M., Strandberg, R., Klaassen, R.H., Tottrup, A.P., Vardanis, Y., Howey, P.W., Thorup, K., Wikelski, M., and Alerstam, T. (2014). Narrow-front loop migration in a population of the common cuckoo Cuculus canorus, as revealed by satellite telemetry. PLoS One *9*, e83515.
20. Hewson, C.M., Thorup, K., Pearce-Higgins, J.W., and Atkinson, P.W. (2016). Population decline is linked to migration route in the Common Cuckoo. Nat. Commun. *7*, 12296. https://doi.org/10.1371/journal.pone.0083515
21. Strandberg, R., Klaassen, R.H., Hake, M., Olofsson, P., and Alerstam, T. (2009). Converging migration routes of Eurasian hobbies Falco subbuteo crossing the African equatorial rain forest. Proc. Biol. Sci. *276*, 727-733. https://doi.org/10.1098/rspb.2008.1202
22. Vansteelant, W.M.G., Gangoso, L., Bouten, W., Viana, D.S., and Figuerola, J. (2021). Adaptive drift and barrier-avoidance by a fly-forage migrant along a climate-driven flyway. Mov. Ecol. *9*, 37. https://doi.org/10.1186/s40462-021-00272-8
23. AlJahdhami, M.H., Karelus, D.L., AlFazari, W.A., de Roland, L.-A.R., Razafimanjato, G., McGrady, M.J., and Oli, M.K. (2021). Movement patterns of Sooty Falcons Falco concolor tracked via satellite across their annual cycle. Bird Study *67*, 505-520. https://doi.org/10.1080/00063657.2021.1950123
24. Gu, Z., Pan, S., Lin, Z., Hu, L., Dai, X., Chang, J., Xue, Y., Su, H., Long, J., Sun, M., et al. (2021). Climate-driven flyway changes and memory-based long-distance migration. Nature 591, 259-264. https://doi.org/10.1038/s41586-021-03265-0
25. Heim, W., Heim, R.J., Beermann, I., Burkovskiy, O.A., Gerasimov, Y., Ktitorov, P., Ozaki, K., Panov, I., Sander, M.M., Sjöberg, S., et al. (2020). Using geolocator tracking data and ringing archives to validate citizen-science based seasonal predictions of bird distribution in a data-poor region. Glob. Ecol. and Conserv. *24*. https://doi.org/10.1016/j.gecco.2020.e01215
26. Klvaňa, P., Cepák, J., Munclinger, P., Michálková, R., Tomášek, O., and Albrecht, T. (2018). Around the Mediterranean: an extreme example of loop migration in a long-distance migratory passerine. J. Avian Biol. *49*. https://doi.org/10.1111/jav.01595
27. Turbek, S.P., Schield, D.R., Scordato, E.S.C., Contina, A., Da, X.W., Liu, Y., Liu, Y., Pagani-Nunez, E., Ren, Q.M., Smith, C.C.R., et al. (2022). A migratory divide spanning two continents is associated with genomic and ecological divergence. Evolution *76*, 722-736. https://doi.org/10.1111/evo.14448
28. Lislevand, T., Chutný, B., Byrkjedal, I., Pavel, V., Briedis, M., Adamik, P., and Hahn, S. (2015). Red-spotted BluethroatsLuscinia s. svecicamigrate along the Indo-European flyway: a geolocator study. Bird Study *62*, 508-515. https://doi.org/10.1080/00063657.2015.1077781
29. Bensch, S., Sokolovskis, K., Willemoes, M., Ivanov, S., Vartanyan, S., and Solovyeva, D. (2022). Geolocator tagging of east Siberian Bluethroats. J. Ornithol. *163*, 843-847. https://doi.org/10.1007/s10336-022-01988-z
30. Briedis, M., Hahn, S., Gustafsson, L., Henshaw, I., Träff, J., Král, M., and Adamík, P. (2016). Breeding latitude leads to different temporal but not spatial organization of the annual cycle in a long-distance migrant. J. Avian Biol. *47*, 743-748. https://doi.org/10.1111/jav.01002
31. Ouwehand, J., Ahola, M.P., Ausems, A.N.M.A., Bridge, E.S., Burgess, M., Hahn, S., Hewson, C.M., Klaassen, R.H.G., Laaksonen, T., Lampe, H.M., et al. (2016). Light-level geolocators reveal

migratory connectivity in European populations of pied flycatchers Ficedula hypoleuca. J. Avian Biol. *47*, 69-83. https://doi.org/10.1111/jav.00721
32. Bell, F., Bearhop, S., Briedis, M., El Harouchi, M., Bell, S.C., Castello, J., and Burgess, M. (2021). Geolocators reveal variation and sex-specific differences in the migratory strategies of a long-distance migrant. Ibis. https://doi.org/10.1111/ibi.13017
33. Briedis, M., Träff, J., Hahn, S., Ilieva, M., Král, M., Peev, S., and Adamík, P. (2016). Year-round spatiotemporal distribution of the enigmatic Semi-collared Flycatcher Ficedula semitorquata. J. Ornithol. *157*, 895-900. https://doi.org/10.1007/s10336-016-1334-6
34. Stach, R., Jakobsson, S., Kullberg, C., and Fransson, T. (2012). Geolocators reveal three consecutive wintering areas in the thrush nightingale. Anim. Migr. *1*, 1-7. https://doi.org/10.2478/ami-2012-0001
35. Emmenegger, T., Hahn, S., and Bauer, S. (2014). Individual migration timing of common nightingales is tuned with vegetation and prey phenology at breeding sites. BMC Ecol. *14*, 9. https://doi.org/10.1186/1472-6785-14-9
36. Bairlein, F., Norris, D.R., Nagel, R., Bulte, M., Voigt, C.C., Fox, J.W., Hussell, D.J., and Schmaljohann, H. (2012). Cross-hemisphere migration of a 25 g songbird. Biol. Lett. *8*, 505-507. https://doi.org/10.1098/rsbl.2011.1223
37. Hiemer, D., Salewski, V., Fiedler, W., Hahn, S., and Lisovski, S. (2017). First tracks of individual Blackcaps suggest a complex migration pattern. J. Ornithol. *159*, 205-210. https://doi.org/10.1007/s10336-017-1490-3
38. Delmore, K.E., Van Doren, B.M., Conway, G.J., Curk, T., Garrido-Garduno, T., Germain, R.R., Hasselmann, T., Hiemer, D., van der Jeugd, H.P., Justen, H., et al. (2020). Individual variability and versatility in an eco-evolutionary model of avian migration. Proc. Biol. Sci. *287*, 20201339. https://doi.org/10.1098/rspb.2020.1339
39. Koleček, J., Procházka, P., El-Arabany, N., Tarka, M., Ilieva, M., Hahn, S., Honza, M., de la Puente, J., Bermejo, A., Gürsoy, A., et al. (2016). Cross-continental migratory connectivity and spatiotemporal migratory patterns in the great reed warbler. J. Avian Biol. *47*, 756-767. https://doi.org/10.1111/jav.00929
40. Procházka, P., Brlík, V., Yohannes, E., Meister, B., Auerswald, J., Ilieva, M., and Hahn, S. (2018). Across a migratory divide: divergent migration directions and non-breeding grounds of Eurasian reed warblers revealed by geolocators and stable isotopes. J. Avian Biol. *49*. https://doi.org/10.1111/jav.00929
41. Salewski, V., Flade, M., Poluda, A., Kiljan, G., Liechti, F., Lisovski, S., and Hahn, S. (2012). An unknown migration route of the 'globally threatened' Aquatic Warbler revealed by geolocators. J. Ornithol. *154*, 549-552. https://doi.org/10.1007/s10336-012-0912-5
42. Tøttrup, A.P., Pedersen, L., and Thorup, K. (2018). Autumn migration and wintering site of a wood warbler Phylloscopus sibilatrix breeding in Denmark identified using geolocation. Anim. Biotelemetry *6*. https://doi.org/10.1186/s40317-018-0159-x
43. Lerche-Jorgensen, M., Willemoes, M., Tottrup, A.P., Snell, K.R.S., and Thorup, K. (2017). No apparent gain from continuing migration for more than 3000 kilometres: willow warblers breeding in Denmark winter across the entire northern Savannah as revealed by geolocators. Mov. Ecol. *5*, 17. https://doi.org/10.1186/s40462-017-0109-x
44. Sokolovskis, K., Bianco, G., Willemoes, M., Solovyeva, D., Bensch, S., and Åkesson, S. (2018). Ten grams and 13,000 km on the wing - route choice in willow warblers Phylloscopus trochilus yakutensis migrating from Far East Russia to East Africa. Mov. Ecol. *6*, 20. https://doi.org/10.1186/s40462-018-0138-0
45. Tottrup, A.P., Klaassen, R.H., Strandberg, R., Thorup, K., Kristensen, M.W., Jorgensen, P.S., Fox, J., Afanasyev, V., Rahbek, C., and Alerstam, T. (2012). The annual cycle of a trans-equatorial

Eurasian-African passerine migrant: different spatio-temporal strategies for autumn and spring migration. Proc. Biol. Sci. *279*, 1008-1016. https://doi.org/10.1098/rspb.2011.1323
46. Thorup, K., Tottrup, A.P., Willemoes, M., Klaassen, R.H., Strandberg, R., Vega, M.L., Dasari, H.P., Araujo, M.B., Wikelski, M., and Rahbek, C. (2017). Resource tracking within and across continents in long-distance bird migrants. Sci. Adv. *3*, e1601360. https://doi.org/10.1126/sciadv.1601360
47. Jiguet, F., Robert, A., Lorrilliere, R., Hobson, K.A., Kardynal, K.J., Arlettaz, R., Bairlein, F., Belik, V., Bernardy, P., Copete, J.L., et al. (2019). Unravelling migration connectivity reveals unsustainable hunting of the declining ortolan bunting. Sci. Adv. *5*, eaau2642. https://doi.org/10.1126/sciadv.aau2642

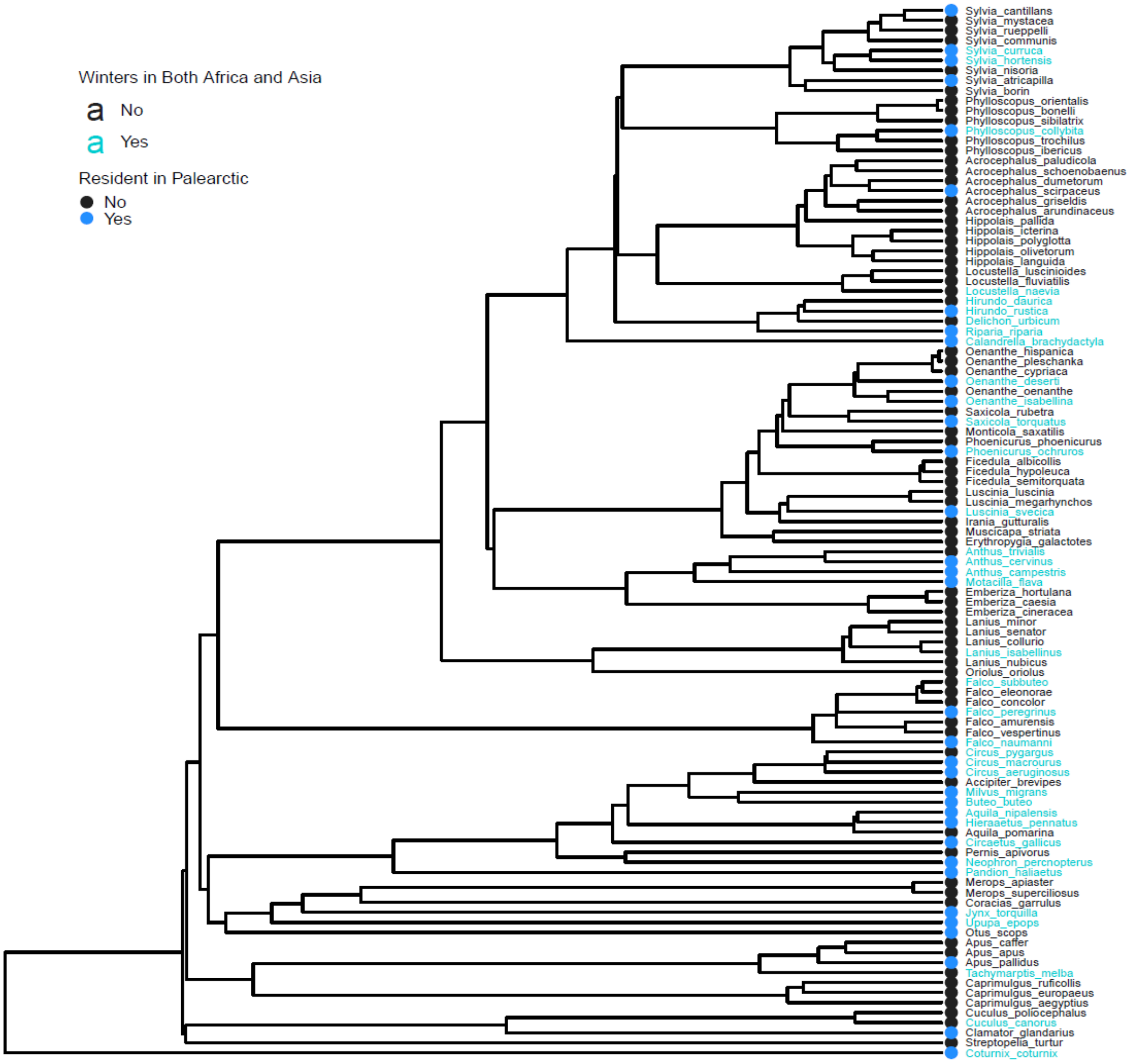

Figure S3. The phylogeny of bird species with tropical wintering ranges restricted to Africa (names in black) and including both Africa and Asia (names in light blue). Related to STAR Methods. Circles indicate species that have (blue) or do not have (black) sedentary or wintering populations in the Palearctic.

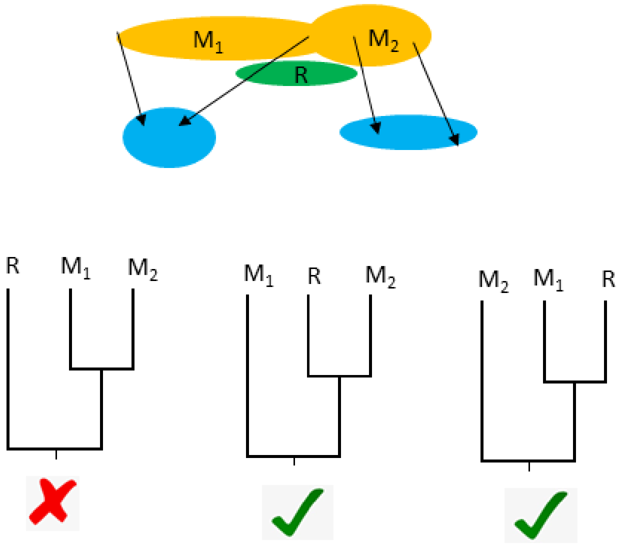

Figure S4. Phylogenetic relationships between migratory and resident populations in support of Models 1 and 2. Related to Figure 1. A hypothetical example of a species having two populations of long-distance migrants (M_1 and M_2) wintering in different tropical regions and one resident population (R). Below are three alternative phylogenetic relationships of which one would reject the suggested model of winter range shifts as illustrated in Figure 1.

Table S1. List of all species included in the analyses with associated trait values. Related to STAR Methods.

Supplementary Table 1: Data on migratory routes, ranges and residency of Palearctic birds

Order	Passerine	Family	SpeciesNames	BirdTreeName	TipName	Migrates.to.Africa.and.Asia	B_Wlong	B_Elong	B_Nlat	B_Slat	W_Wlon	W_Elong	W_Nlat	W_Slat	ResidentShort	MinLongD	NoSubsp	Subspecies.wintering.in.both.Africa.and.Asia	TaxonomyComment
Accipitriformes	0	Accipitridae	Accipiter brevipes	Accipiter brevipes	Accipiter_brevipes	0	17	51	52	28	23	40	19	1	0	4400	1		
Accipitriformes	0	Accipitridae	Buteo buteo	Buteo buteo	Buteo_buteo	1	-28	99	68	28	-28	77	59	-35	1	1800	7	vulpinis	
Accipitriformes	0	Accipitridae	Circaetus gallicus	Circaetus gallicus	Circaetus_gallicus	1	-10	112	60	18	15	96	36	5	1	5100	1	gallicus	
Accipitriformes	0	Accipitridae	Circus aeruginosus	Circus aeruginosus	Circus_aeruginosus	1	-9	104	66	28	-25	94	52	-26	1	3500	2	aeruginosus	
Accipitriformes	0	Accipitridae	Circus macrourus	Circus macrourus	Circus_macrourus	1	28	103	56	41	-17	97	37	-34	1	3500	1	macrourus	
Accipitriformes	0	Accipitridae	Circus pygargus	Circus pygargus	Circus_pygargus	1	-9	91	61	31	-17	88	34	-32	0	4800	1	pygargus	
Accipitriformes	0	Accipitridae	Aquila nipalensis	Aquila nipalensis	Aquila_nipalensis	1	33	120	50	29	13	112	36	-26	1	3200	2		
Accipitriformes	0	Accipitridae	Aquila pomarina	Aquila pomarina	Aquila_pomarina	0	10	55	59	35	14	41	21	-27	0	4200	1		
Accipitriformes	0	Accipitridae	Hieraaetus pennatus	Hieraaetus pennatus	Hieraaetus_pennatus	1	-9	121	58	30	-17	92	33	-35	1	3600	1	pennatus	
Accipitriformes	0	Accipitridae	Milvus migrans	Milvus migrans	Milvus_migrans	1	-17	142	71	28	-17	146	43	-39	1	3300	7		
Accipitriformes	0	Accipitridae	Neophron percnopterus	Neophron percnopterus	Neophron_percnopterus	1	-25	87	49	25	-25	87	32	-3	1	2000	3	percnopterus	
Accipitriformes	0	Accipitridae	Pernis apivorus	Pernis apivorus	Pernis_apivorus	0	-6	88	67	35	-17	45	14	-34	0	7000	1		
Accipitriformes	0	Pandionidae	Pandion haliaetus	Pandion haliaetus	Pandion_haliaetus	1	-25	165	69	15	-25	156	49	-35	1	3400	3	haliaetus	
Galliformes	0	Phasianidae	Coturnix coturnix	Coturnix coturnix	Coturnix_coturnix	1	-12	110	63	23	-17	93	37	-33	1	4000	4	coturnix	
Columbiformes	0	Columbidae	Streptopelia turtur	Streptopelia turtur	Streptopelia_turtur	0	-18	96	63	15	-17	41	17	5	0	6000	4		
Caprimulgiformes	0	Apodidae	Apus apus	Apus apus	Apus_apus	0	-11	122	69	33	-12	41	10	-31	0	10100	2		
Caprimulgiformes	0	Apodidae	Apus pallidus	Apus pallidus	Apus_pallidus	0	-17	59	46	17	-17	65	35	5	1	3500	3		
Caprimulgiformes	0	Apodidae	Tachymarptis melba	Tachymarptis melba	Tachymarptis_melba	1	-9	88	47	26	-11	81	18	-28	0	3300	10		
Caprimulgiformes	0	Apodidae	Apus caffer	Apus caffer	Apus_caffer	0	-8	-3	39	30	-17	48	16	-25	0	2800	1		
Caprimulgiformes	0	Caprimulgidae	Caprimulgus aegyptius	Caprimulgus aegyptius	Caprimulgus_aegyptius	0	-12	73	49	22	-17	33	19	12	0	4700	2		
Caprimulgiformes	0	Caprimulgidae	Caprimulgus europaeus	Caprimulgus europaeus	Caprimulgus_europaeus	0	-9	119	63	24	-17	40	16	-35	0	9000	6		
Caprimulgiformes	0	Caprimulgidae	Caprimulgus ruficollis	Caprimulgus ruficollis	Caprimulgus_ruficollis	0	-10	11	42	30	-17	-2	22	12	0	2700	2		
Coraciiformes	0	Coraciidae	Coracias garrulus	Coracias garrulus	Coracias_garrulus	0	-9	82	59	32	-6	46	6	-32	0	6600	2		
Coraciiformes	0	Meropidae	Merops apiaster	Merops apiaster	Merops_apiaster	0	-9	82	57	29	-17	41	17	-34	0	6200	1		
Coraciiformes	0	Meropidae	Merops persicus	Merops superciliosus	Merops_persicus	0	-10	80	47	28	-17	50	15	-26	0	2500	2		
Coraciiformes	0	Upupidae	Upupa epops	Upupa epops	Upupa_epops	1	-16	141	60	28	-17	47	18	-34	1	3800	7	epops	
Cuculiformes	0	Cuculidae	Clamator glandarius	Clamator glandarius	Clamator_glandarius	0	-9	46	46	28	-17	48	35	-17	1	1400	1		
Cuculiformes	0	Cuculidae	Cuculus canorus	Cuculus canorus	Cuculus_canorus	1	-9	177	73	20	-17	107	21	-35	0	6700	4	canorus, subtelephonus	
Cuculiformes	0	Cuculidae	Cuculus poliocephalus	Cuculus poliocephalus	Cuculus_poliocephalus	0	71	142	42	20	24	40	-3	-24	0	11300	1		
Strigiformes	0	Strigidae	Otus scops	Otus scops	Otus_scops	0	-10	108	58	31	-17	50	18	0	1	7300	5		
Piciformes	0	Picidae	Jynx torquilla	Jynx torquilla	Jynx_torquilla	1	-9	143	69	35	-16	43	18	5	1	3400	6	torquilla	
Falconiformes	0	Falconidae	Falco naumanni	Falco naumanni	Falco_naumanni	1	-9	121	58	27	-17	100	26	-35	1	3600	1	naumanni	
Falconiformes	0	Falconidae	Falco subbuteo	Falco subbuteo	Falco_subbuteo	1	-9	170	67	21	-12	116	28	-34	0	6200	2	subbuteo	
Falconiformes	0	Falconidae	Falco vespertinus	Falco vespertinus	Falco_vespertinus	0	19	127	64	41	14	30	-9	-30	0	11000	1		
Falconiformes	0	Falconidae	Falco amurensis	Falco amurensis	Falco_amurensis	0	115	139	54	37	14	34	-29	-34	0	12000	1		
Falconiformes	0	Falconidae	Falco eleonorae	Falco eleonorae	Falco_eleonorae	0	-14	34	45	28	33	50	-13	-25	0	7200	1		
Falconiformes	0	Falconidae	Falco concolor	Falco concolor	Falco_concolor	0	20	66	32	15	33	50	-12	-26	0	4600	1		
Falconiformes	0	Falconidae	Falco peregrinus	Falco peregrinus	Falco_peregrinus	1	-23	190	80	15	-23	179	60	-34	1	3900	19	peregrinus, calidus	
Passeriformes	1	Alaudidae	Calandrella brachydactyla	Calandrella brachydactyla	Calandrella_brachydactyla	1	-16	96	54	20	-17	78	48	1	1	3300	7		
Passeriformes	1	Hirundinidae	Delichon urbicum	Delichon urbicum	Delichon_urbicum	1	-10	179	70	29	-17	107	14	-34	0	6400	3	meridionale	
Passeriformes	1	Hirundinidae	Cecropis daurica	Hirundo daurica	Cecropis_daurica	1	-10	140	53	14	-17	145	26	-19	0	4500	9	rufula	
Passeriformes	1	Hirundinidae	Hirundo rustica	Hirundo rustica	Hirundo_rustica	1	-10	179	70	21	-17	151	38	-34	1	6300	7	rustica	
Passeriformes	1	Hirundinidae	Riparia riparia	Riparia riparia	Riparia_riparia	1	-10	174	71	27	-17	91	30	-28	1	7200	4		
Passeriformes	1	Motacillidae	Anthus campestris	Anthus campestris	Anthus_campestris	1	-9	98	60	27	-17	80	33	-2	1	3200	1	campestris	
Passeriformes	1	Motacillidae	Anthus trivialis	Anthus trivialis	Anthus_trivialis	1	-9	130	70	30	-17	87	28	-23	0	6200	2	trivialis	
Passeriformes	1	Motacillidae	Anthus cervinus	Anthus cervinus	Anthus_cervinus	1	15	190	75	49	-17	121	37	-5	1	6000	1	cervinus	
Passeriformes	1	Motacillidae	Motacilla flava	Motacilla flava	Motacilla_flava	1	-9	128	71	17	-17	94	35	-34	1	5500	10	lutea, thunbergi, beema, feldegg	
Passeriformes	1	Muscicapidae	Erythropygia galactotes	Erythropygia galactotes	Erythropygia_galactotes	0	-16	76	47	25	-17	51	19	-4	0	4500	5		
Passeriformes	1	Muscicapidae	Irania gutturalis	Irania gutturalis	Irania_gutturalis	0	29	71	41	27	35	40	8	-5	0	4900	1		
Passeriformes	1	Muscicapidae	Luscinia svecica	Luscinia svecica	Luscinia_svecica	1	-8	225	71	33	-17	121	43	8	1	7700	12	svecica	
Passeriformes	1	Muscicapidae	Ficedula albicollis	Ficedula albicollis	Ficedula_albicollis	0	6	57	57	37	23	35	-9	-21	0	7500	1		
Passeriformes	1	Muscicapidae	Ficedula hypoleuca	Ficedula hypoleuca	Ficedula_hypoleuca	0	-6	93	71	32	-17	30	13	-7	0	8300	4		F. speculigera included
Passeriformes	1	Muscicapidae	Ficedula semitorquata	Ficedula semitorquata	Ficedula_semitorquata	0	20	53	45	35	28	36	4	-9	0	4800	1		
Passeriformes	1	Muscicapidae	Luscinia luscinia	Luscinia luscinia	Luscinia_luscinia	0	5	94	65	46	22	35	-8	-26	0	8700	1		
Passeriformes	1	Muscicapidae	Luscinia megarhynchos	Luscinia megarhynchos	Luscinia_megarhynchos	0	-9	92	54	24	-17	44	15	-5	0	6500	3		
Passeriformes	1	Muscicapidae	Monticola saxatilis	Monticola saxatilis	Monticola_saxatilis	0	-9	119	49	29	-17	42	18	-10	0	8100	1		
Passeriformes	1	Muscicapidae	Muscicapa striata	Muscicapa striata	Muscicapa_striata	0	-10	116	70	25	-17	47	13	-34	0	8500	5		
Passeriformes	1	Muscicapidae	Oenanthe hispanica	Oenanthe hispanica	Oenanthe_hispanica	0	-9	58	46	28	-17	43	22	12	0	3000	2		
Passeriformes	1	Muscicapidae	Oenanthe oenanthe	Oenanthe oenanthe	Oenanthe_oenanthe	0	-90	234	76	30	-17	45	19	-15	0	12000	4		O. seebohmi included
Passeriformes	1	Muscicapidae	Oenanthe deserti	Oenanthe deserti	Oenanthe_deserti	1	-17	115	52	23	-17	83	35	1	1	3700	3		
Passeriformes	1	Muscicapidae	Oenanthe isabellina	Oenanthe isabellina	Oenanthe_isabellina	1	23	117	51	29	-16	85	40	-3	1	3600	1	isabellina	
Passeriformes	1	Muscicapidae	Oenanthe pleschanka	Oenanthe pleschanka	Oenanthe_pleschanka	0	27	75	52	32	30	43	19	-7	0	7100	1		
Passeriformes	1	Muscicapidae	Oenanthe cypriaca	Oenanthe cypriaca	Oenanthe_cypriaca	0	34	36	36	34	13	39	20	10	0	1800	1		
Passeriformes	1	Muscicapidae	Phoenicurus phoenicurus	Phoenicurus phoenicurus	Phoenicurus_phoenicurus	0	-9	113	70	27	-17	50	18	-1	0	6700	2		
Passeriformes	1	Muscicapidae	Phoenicurus ochruros	Phoenicurus ochruros	Phoenicurus_ochruros	1	-9	117	61	29	-16	98	54	7	1	3100	7	phoenicuroides	
Passeriformes	1	Muscicapidae	Saxicola rubetra	Saxicola rubetra	Saxicola_rubetra	0	-10	96	69	36	-17	46	16	-14	0	6100	1		
Passeriformes	1	Muscicapidae	Saxicola maurus	Saxicola torquatus	Saxicola_maurus	1	34	112	67	18	29	102	37	8	1	6100	5	variegatus	
Passeriformes	1	Sylviidae	Sylvia atricapilla	Sylvia atricapilla	Sylvia_atricapilla	0	-28	62	68	14	-28	43	56	-10	1	4200	5		
Passeriformes	1	Sylviidae	Sylvia borin	Sylvia borin	Sylvia_borin	0	-10	90	70	39	-17	38	14	-32	0	7700	2		
Passeriformes	1	Sylviidae	Sylvia cantillans	Sylvia cantillans	Sylvia_cantillans	0	-10	33	46	27	-17	34	31	12	1	2400	5		S. inornata and S. subalpina included
Passeriformes	1	Sylviidae	Sylvia communis	Sylvia communis	Sylvia_communis	0	-10	104	68	31	-17	45	16	-26	0	6900	4		
Passeriformes	1	Sylviidae	Sylvia curruca	Sylvia curruca	Sylvia_curruca	1	-4	124	68	30	-17	82	29	7	1	5000	6		
Passeriformes	1	Sylviidae	Sylvia hortensis	Sylvia hortensis	Sylvia_hortensis	1	-11	81	47	26	-17	84	29	9	1	3100	5		S. crassirostris included
Passeriformes	1	Sylviidae	Sylvia nisoria	Sylvia nisoria	Sylvia_nisoria	0	8	98	62	35	32	41	5	-5	0	6600	2		
Passeriformes	1	Sylviidae	Sylvia rueppelli	Sylvia rueppelli	Sylvia_rueppelli	0	20	37	41	35	7	37	32	12	0	2100	1		
Passeriformes	1	Sylviidae	Sylvia mystacea	Sylvia mystacea	Sylvia_mystacea	0	36	72	47	30	33	59	28	9	0	2000	3		
Passeriformes	1	Acrocephalidae	Acrocephalus arundinaceus	Acrocephalus arundinaceus	Acrocephalus_arundinaceus	0	-9	93	61	32	-17	40	14	-30	0	7000	2		
Passeriformes	1	Acrocephalidae	Acrocephalus griseldis	Acrocephalus griseldis	Acrocephalus_griseldis	0	34	48	33	28	27	44	2	-25	0	3300	1		
Passeriformes	1	Acrocephalidae	Acrocephalus palustris	Acrocephalus dumetorum	Acrocephalus_palustris	0	-2	61	64	37	25	40	-9	-33	0	7600	1		
Passeriformes	1	Acrocephalidae	Acrocephalus schoenobaenus	Acrocephalus schoenobaenus	Acrocephalus_schoenobaenus	0	-10	84	70	37	-17	44	17	-32	0	7000	1		
Passeriformes	1	Acrocephalidae	Acrocephalus scirpaceus	Acrocephalus scirpaceus	Acrocephalus_scirpaceus	0	-10	86	64	26	-17	41	16	-26	1	5600	4		
Passeriformes	1	Acrocephalidae	Acrocephalus paludicola	Acrocephalus paludicola	Acrocephalus_paludicola	0	13	39	56	47	-17	1	20	13	0	5000	1		
Passeriformes	1	Acrocephalidae	Hippolais icterina	Hippolais icterina	Hippolais_icterina	0	2	87	68	36	13	37	0	-32	0	7500	1		
Passeriformes	1	Acrocephalidae	Hippolais polyglotta	Hippolais polyglotta	Hippolais_polyglotta	0	-9	18	51	30	-17	15	12	4	0	4300	1		
Passeriformes	1	Acrocephalidae	Iduna languida	Hippolais languida	Iduna_languida	0	33	74	46	25	33	51	18	-7	0	3800	1		
Passeriformes	1	Acrocephalidae	Iduna olivetorum	Hippolais olivetorum	Iduna_olivetorum	0	16	40	44	42	17	38	0	-30	0	5200	1		

Passeriformes	1	Acrocephalidae	Iduna pallida	Hippolais pallida	Iduna_pallida	0	-14	82	49	15	-17	48	20	-7	0	4900	4		I. opaca included
Passeriformes	1	Locustellidae	Locustella fluviatilis	Locustella fluviatilis	Locustella_fluviatilis	0	8	76	63	42	21	35	-13	-25	0	8000	1		
Passeriformes	1	Locustellidae	Locustella luscinioides	Locustella luscinioides	Locustella_luscinioides	0	-9	86	58	33	-17	42	18	5	0	5900	3		
Passeriformes	1	Locustellidae	Locustella naevia	Locustella naevia	Locustella_naevia	1	-10	98	65	39	-17	78	24	9	0	5900	4	straminea	
Passeriformes	1	Phylloscopidae	Phylloscopus bonelli	Phylloscopus bonelli	Phylloscopus_bonelli	0	-9	17	50	30	-17	22	16	10	0	3800	1		
Passeriformes	1	Phylloscopidae	Phylloscopus orientalis	Phylloscopus orientalis	Phylloscopus_orientalis	0	16	46	45	34	22	39	21	9	0	3500	1		
Passeriformes	1	Phylloscopidae	Phylloscopus collybita	Phylloscopus collybita	Phylloscopus_collybita	1	-10	161	72	36	-17	93	52	-1	1	6500	6		
Passeriformes	1	Phylloscopidae	Phylloscopus ibericus	Phylloscopus ibericus	Phylloscopus_ibericus	0	-9	0	43	33	-17	14	15	11	0	3200	1		
Passeriformes	1	Phylloscopidae	Phylloscopus sibilatrix	Phylloscopus sibilatrix	Phylloscopus_sibilatrix	0	-10	65	68	38	-16	45	16	-6	0	6200	1		
Passeriformes	1	Phylloscopidae	Phylloscopus trochilus	Phylloscopus trochilus	Phylloscopus_trochilus	0	-10	178	72	43	-17	41	15	-34	0	11000	3		
Passeriformes	1	Laniidae	Lanius collurio	Lanius collurio	Lanius_collurio	0	-9	91	65	35	14	51	17	-34	0	6600	1		
Passeriformes	1	Laniidae	Lanius minor	Lanius minor	Lanius_minor	0	1	89	56	30	13	33	-17	-32	0	10000	2		
Passeriformes	1	Laniidae	Lanius senator	Lanius senator	Lanius_senator	0	-9	59	53	28	-17	42	18	-3	0	4100	4		
Passeriformes	1	Laniidae	Lanius nubicus	Lanius nubicus	Lanius_nubicus	0	22	52	42	28	-8	45	19	8	0	2300	1		
Passeriformes	1	Laniidae	Lanius isabellinus	Lanius isabellinus	Lanius_isabellinus	1	49	120	53	26	-17	88	33	-5	0	5000	4		L. phoenicuroides included
Passeriformes	1	Oriolidae	Oriolus oriolus	Oriolus oriolus	Oriolus_oriolus	0	-9	99	62	29	13	40	7	-34	0	8200	1		
Passeriformes	1	Emberizidae	Emberiza hortulana	Emberiza hortulana	Emberiza_hortulana	0	-9	104	68	36	-17	38	21	6	0	6900	1		
Passeriformes	1	Emberizidae	Emberiza caesia	Emberiza caesia	Emberiza_caesia	0	19	42	41	31	29	39	19	13	0	2600	1		
Passeriformes	1	Emberizidae	Emberiza cineracea	Emberiza cineracea	Emberiza_cineracea	0	26	46	40	35	37	44	21	13	0	2100	2		

Table S2: Bayesian Phylogenetic Mixed Model of the effect of eastern breeding range on the probability of wintering in Asia.Parameter estimates (posterior modes and 95% credible intervales) are on the logit scale. Related to Figure 3.

**Fixed Effects**	**Posterior Mode (CI)**	**pMCMC**
Intercept	-1.46 (-175.62, -0.19)	**0.001**
Eastern longitude	2.78 (0.95, 265.11)	**0.001**
**Random Effects**	**Posterior Mode (CI)**	**I2 % (CI)**
Phylogeny	-74.05 (0, 50616.82)	35.38 (0, 99.99)
Residual	-62.53 (0, 72097.31)	60.12 (0, 99.99)

Table S3. Bayesian Phylogenetic Mixed Model of the effect of migration distance and the presence of resident populations on the probability that species migrate to Africa and Asia (binary response variable). Related to Figure 3.

Model includes western and eastern longitude, and southern latitude of breeding ranges. Northern latitude was not included as it was strongly correlated to eastern longitude (r = 0.7).

**Fixed Effects**	**Posterior Mode (CI)**	**pMCMC**
Intercept	-2.84 (-210.25, -1.98)	**0.001**
Resident population	3.38 (-5.51, 218)	**0.038**
Minimum longest migration distance (km)	-187.43 (-271.09, -2.93)	**0.001**
Western longitude	1.09 (-16.35, 69.75)	0.206
Eastern longitude	274.41 (4.89, 344.43)	**0.001**
Southern latitude	-0.18 (-39.52, 57.1)	0.724
**Random Effects**	**Posterior Mode (CI)**	**I2 % (CI)**
Phylogeny	-24.9 (0, 14213.53)	37.48 (0, 99.96)
Residual	73.76 (0, 18231.47)	59.66 (0, 99.98)

Table S4: The same analysis as presented in Table S3, but including northern latitude instead of eastern longitude. Related to Figure 3. This was to verify that the way breeding range variation was controlled for does not influence the outcome of the analysis.

**Fixed Effects**	**Posterior Mode (CI)**	**pMCMC**
Intercept	-187.91 (-364.28, -60.56)	**0.001**
Resident population	235.08 (2.08, 372.91)	**0.004**
Minimum longest migration distance (km)	-147.33 (-340.78, -44.26)	**0.001**
Western longitude	72.53 (-0.35, 177.77)	0.054
Northern latitude	238.14 (83.53, 374.9)	**0.001**
Southern latitude	-48.88 (-119.25, 11.92)	0.112
**Random Effects**	**Posterior Mode (CI)**	**I2 % (CI)**
Phylogeny	44.73 (0, 48403.58)	40.6 (0, 99.99)
Residual	-19.49 (0, 54063.74)	58.72 (0, 99.99)

```r
#******************************************************************************
# Analysis script by Charlie Cornwallis
#******************************************************************************
#The response variable is: BothAfAs - Winters in both Africa and Asia (0=No, 1=Yes).

#The main two predictor variables are:
#ResidentShort  - Whether the species has resident or short distance migrants in the Palearctic (0=No, 1=Yes)
#MinLongD – The minimum longest migration distance (Range 1400-12000 km).

#The model should control for the endpoints of the breeding range:
#B_Wlong - Breeding West Longitude
#B_Elong - Breeding East Longitude
#B_Nlat - Breeding North Latitude
#B_Slat - Breeding South Latitude

#I have collected estimates of migration distance from about 35 species (longest distance by satellite tracking of geolocation) and there is a nice agreement with our proxy for migration distance – MinLongD (attached Fig).
#I have not checked whether MinLongD is correlated with 'W_Slat'  (Winter South Latitude) but it might also be a proxy of migratoriness so also worth testing as a response variable.

#******************************************************************************
# Packages, Data and Settings ####
#******************************************************************************
pacman::p_load("MCMCglmm","dplyr","tidyr")
load("./Data/Data.RData")

#******************************************************************************
#Function for tree sampling ####
#******************************************************************************
MCMCglmm_trees<-function(prior, data, trees,tiplabel,fixed,random,Nrandom,rcov=as.formula(~units),mev=NULL,family="gaussian",samples=1000,nitts,thins,burns){
#1. Create object to write model output to
tree1<-inverseA(trees[[1]])$Ainv
data$tiplabel<-tiplabel

model<-MCMCglmm(fixed=fixed, random=random,
                ginverse=list(tiplabel=tree1),family =family, data = data,prior=prior,
                nitt=samples, burnin=0, thin=1,pr=TRUE,verbose = F,pl=T)
modeltmp<-model

#2. Run model on each tree. Estimates of last tree provide start values for next tree. Use first 500 trees as a burnin.
for(i in 1:length(trees)){
  tree<-trees[[i]]
  invtree<-inverseA(tree)$Ainv

  #Setup starting values
  tmp <- list(Liab=modeltmp$Liab[1,], R=modeltmp$VCV[1,Nrandom+1])
  Gtmp=list()
  for(j in 1:Nrandom){
```

```
      G1=list(modeltmp$VCV[1,j])
      names(G1)<-paste("G",j,sep="")
      Gtmp=c(Gtmp,G1)
    }
    G=list(Gtmp)
    names(G)<-"G"
    start=c(tmp,G)

    modeltmp<-MCMCglmm(fixed=fixed, random=random,
                       ginverse=list(tiplabel=invtree),family =family, data = data,prior=prior,
                       nitt=nitts, thin=thins,burnin=burns, start=start, pr=TRUE,verbose = F,pl=T)
    if(i>500){
      model$VCV[i-500,]<-modeltmp$VCV[1,]
      model$Sol[i-500,]<-modeltmp$Sol[1,]
      model$Liab[i-500,]<-modeltmp$Liab[1,]
    }
    #print(i)
  }
  return(model)
  }

  #*************************************************************************************
  #Settings ####
  #*************************************************************************************
  #Checks
  nitts=10
  burns=9
  thins=1

  #Full runs: 1000 trees = 10 million iterations with a 500 tree / 5 million iteration burnin
  nitts=10000
  burns=9999
  thins=1

  #*************************************************************************************
  #Analysis 1: Effect of eastern breeding range (continuous fixed effect) on the probability of wintering in Asia  ####
  #*************************************************************************************
  m1_1<-MCMCglmm_trees(prior=list(R = list(V = 1,nu=0.002), G = list(G1=list(V = 1, nu = 0.002))),
                                    data=data,
                                    samples=1000,
                                    trees=trees,tiplabel=data$tipname,
                                    fixed=as.formula(BothAfAs ~ ZB_Elong),
                                    random=as.formula(~tipname),
                                                        Nrandom=1,family="categorical",nitts=nitts,thins=thins,burns=burns)

  m1_2<-MCMCglmm_trees(prior=list(R = list(V = 1,nu=0.002), G = list(G1=list(V = 1, nu = 0.002))),
                                    data=data,
                                    samples=1000,
                                    trees=trees,tiplabel=data$tipname,
```

```
                                    fixed=as.formula(BothAfAs ~ ZB_Elong),
                                    random=as.formula(~tipname),
                                                      Nrandom=1,family="categorical",nitts=nitts,thins=thins,burns=burns)

m1_3<-MCMCglmm_trees(prior=list(R = list(V = 1,nu=0.002), G = list(G1=list(V = 1, nu = 0.002))),
                                    data=data,
                                    samples=1000,
                                    trees=trees,tiplabel=data$tipname,
                                    fixed=as.formula(BothAfAs ~ ZB_Elong),
                                    random=as.formula(~tipname),
                                                      Nrandom=1,family="categorical",nitts=nitts,thins=thins,burns=burns)

#Check convergence
m1.Sol<-mcmc.list(m1_1$Sol,m1_2$Sol,m1_3$Sol)
m1.VCV<-mcmc.list(m1_1$VCV,m1_2$VCV,m1_3$VCV)

gelman.diag(m1.Sol,multivariate = FALSE)
gelman.diag(m1.VCV,multivariate = FALSE)

#plot(m1_1)
summary(m1_1)

#*****************************************************************************************
#Analysis 2: Effect of migration distance and the presence of resident populations on the probability that species migrate to Africa and Asia (binary response variable) ####
#*****************************************************************************************
m2_1<-MCMCglmm_trees(prior=list(R = list(V = 1,nu=0.002), G = list(G1=list(V = 1, nu = 0.002))),
                                    data=data,
                                    samples=1000,
                                    trees=trees,tiplabel=data$tipname,
                                    fixed=as.formula(BothAfAs ~ ResidentShort+ZMinLongD+ZB_Wlong+ZB_Elong+ZB_Slat),
                                    random=as.formula(~tipname),
                                                      Nrandom=1,family="categorical",nitts=nitts,thins=thins,burns=burns)

m2_2<-MCMCglmm_trees(prior=list(R = list(V = 1,nu=0.002), G = list(G1=list(V = 1, nu = 0.002))),
                                    data=data,
                                    samples=1000,
                                    trees=trees,tiplabel=data$tipname,
                                    fixed=as.formula(BothAfAs ~ ResidentShort+ZMinLongD+ZB_Wlong+ZB_Elong+ZB_Slat),
                                    random=as.formula(~tipname),
                                                      Nrandom=1,family="categorical",nitts=nitts,thins=thins,burns=burns)

m2_3<-MCMCglmm_trees(prior=list(R = list(V = 1,nu=0.002), G = list(G1=list(V = 1, nu = 0.002))),
                                    data=data,
                                    samples=1000,
                                    trees=trees,tiplabel=data$tipname,
                                    fixed=as.formula(BothAfAs ~ ResidentShort+ZMinLongD+ZB_Wlong+ZB_Elong+ZB_Slat),
                                    random=as.formula(~tipname),
                                                      Nrandom=1,family="categorical",nitts=nitts,thins=thins,burns=burns)
```

```
#Check convergence
m2.Sol<-mcmc.list(m2_1$Sol,m2_2$Sol,m2_3$Sol)
m2.VCV<-mcmc.list(m2_1$VCV,m2_2$VCV,m2_3$VCV)

gelman.diag(m2.Sol,multivariate = FALSE)
gelman.diag(m2.VCV,multivariate = FALSE)

#plot(m2_1)
summary(m2_1)

#**************************************************************************************
#The same analysis as presented in supplementary 2_1, but including northern latitude instead of eastern longitude. This is to verify that the way breeding range variation is controlled for does not influence the outcome of analyses ####
#**************************************************************************************
m3_1<-MCMCglmm_trees(prior=list(R = list(V = 1,nu=0.002), G = list(G1=list(V = 1, nu = 0.002))),
                                    data=data,
                                    samples=1000,
                                    trees=trees,tiplabel=data$tipname,
                                    fixed=as.formula(BothAfAs ~ ResidentShort+ZMinLongD+ZB_Wlong+ZB_Nlat+ZB_Slat),
                                    random=as.formula(~tipname),
                                                      Nrandom=1,family="categorical",nitts=nitts,thins=thins,burns=burns)

m3_2<-MCMCglmm_trees(prior=list(R = list(V = 1,nu=0.002), G = list(G1=list(V = 1, nu = 0.002))),
                                    data=data,
                                    samples=1000,
                                    trees=trees,tiplabel=data$tipname,
                                    fixed=as.formula(BothAfAs ~ ResidentShort+ZMinLongD+ZB_Wlong+ZB_Nlat+ZB_Slat),
                                    random=as.formula(~tipname),
                                                      Nrandom=1,family="categorical",nitts=nitts,thins=thins,burns=burns)

m3_3<-MCMCglmm_trees(prior=list(R = list(V = 1,nu=0.002), G = list(G1=list(V = 1, nu = 0.002))),
                                    data=data,
                                    samples=1000,
                                    trees=trees,tiplabel=data$tipname,
                                    fixed=as.formula(BothAfAs ~ ResidentShort+ZMinLongD+ZB_Wlong+ZB_Nlat+ZB_Slat),
                                    random=as.formula(~tipname),
                                                      Nrandom=1,family="categorical",nitts=nitts,thins=thins,burns=burns)

#Check convergence
m3.Sol<-mcmc.list(m3_1$Sol,m3_2$Sol,m3_3$Sol)
m3.VCV<-mcmc.list(m3_1$VCV,m3_2$VCV,m3_3$VCV)

gelman.diag(m3.Sol,multivariate = FALSE)
gelman.diag(m3.VCV,multivariate = FALSE)

summary(m3_1)
```

```r
#*****************************************************************************************
#Saving outputs
#*****************************************************************************************
save.image("./Results/Models.RData")
```
